# The Influence of Konjac Glucomannan on the Physicochemical and Rheological Properties and Microstructure of Canna Starch

**DOI:** 10.3390/foods10020422

**Published:** 2021-02-15

**Authors:** Yuanqin Liu, Qiaoli Chen, Fang Fang, Jiali Liu, Zhiying Wang, Hourong Chen, Fusheng Zhang

**Affiliations:** 1 College of Food Science, Southwest University, Chongqing 400715, China; qinqin0407@email.swu.edu.cn (Y.L.); cql123@email.swu.edu.cn (Q.C.); minerva233@email.swu.edu.cn (J.L.); wzy1135013@email.edu.cn (Z.W.); chourong@swu.edu.cn (H.C.); 2 Whistler Center for Carbohydrate Research, Department of Food Science, Purdue University, West Lafayette, IN 47906, USA; ffang@purdue.edu

**Keywords:** konjac glucomannan, canna starch, gelatinization property, rheological property, microstructure

## Abstract

The addition of hydrocolloid is an effective method to improve the properties of native starch. However, few studies have investigated the effects of konjac glucomannan (KGM) on canna starch (CS). In this study, the effects of various KGM concentration on the pasting, rheological, textural, and morphological properties of CS were investigated. The addition of KGM significantly increased CS’s pasting viscosities. Incorporation of KGM in CS at a relatively high level (1.2% *w*/*w*) exerted a significant influence on the pasting properties of CS. The consistency coefficient of CS was notably increased by KGM (from 43.6 to 143.3 Pa·s^n^) and positively correlated positive with KGM concentration. KGM concentration at a relatively high level (1.2% *w*/*w*) increased the elasticities and cohesiveness of CS by 53.3% and 88.0%, respectively, in texture profile analysis. The polarized optical microscope images indicated that KGM played an important part in protecting the crystalline structure of CS during heating. A denser porous microstructure with a filamentous network was observed in gelatinized KGM/CS mixtures as compared with the CS control. This research advances the knowledge of interactions between KGM and CS and opens possibilities to improve rheological properties of CS and to develop its new functionalities with KGM addition.

## 1. Introduction

Starch, one of the most important polysaccharides, has been used in food industrial applications for many years due to its low cost, biodegradability, non-toxicity, and processing applicability. The behaviors of its gelatinization are critical factors for starch-based food applications. Starches isolated from corn, wheat, rice, and potato have been extensively studied [1,2,3,4]. Investigating new starch sources is important for improving and developing functionalities that can enrich starch-based products [3]. According to previous reports, canna starch (CS) is a potential candidate for the food industry, but it depends on an abundance of raw material, several minor constituents (calcium, phosphorus, and fluorine), high transparency, easy gelatinization, and digestibility [5,6,7].

*Canna edulis Ker* (CK) is widely planted across many regions, such as South America, Vietnam, Thailand, and China [6,7]. The tuber of CK contains up to 60~80% starch (dry basis), making it a potential starch source for the food industry that has not yet been fully developed and utilized [5]. Native CS usually has a high gelatinization temperature, weak resistance against retrogradation, and gel structure cohesion [8], which limits application development. The modification of native CS for improving its processing applicability, including physical, chemical, biological and combined modifications has been attracting increasing attention.

The addition of hydrocolloids is an economical, effective, and environmentally friendly approach for improving the properties of native starches. Non-starch hydrocolloids have been widely used to enhance the textural and rheological properties of native starch [9,10,11], owing to their high molecular weight and good water binding ability. Furthermore, the gelatinization process of native starch can be altered by hydrocolloids because of their water imbibition [12]. The presence of non-starch hydrocolloids in native starch results in the formation of a stable polymeric network that surrounds starch granules and delays the release of amylose [1,9]. The interactions between starch and hydrocolloid improve textural properties, retard starch retrogradation, increase moisture retention, and contribute to the overall quality of starchy food products [1,13].

Konjac glucomannan (KGM) is a neutral hydrophilic colloid derived from the rhizomes of *Amorphophallus konjac C. Koch* [9]. As a non-ionic water-soluble polysaccharide, it is mainly composed of D-glucose and D-mannose, which are polymerized by β-1,4-glycoside bonds in molecular ratio of 1:1.6. KGM has a high viscosity due to its high molecular weight and intermolecular interactions, thus, providing improved gelling and water holding capacity when used in the food industry [14,15]. Additionally, KGM, a kind of macromolecular polymer with high density and branching structure, interacts with starch and manipulates starch properties. Through incorporation with starch, it can effectively improve the viscoelasticity, reduce starch retrogradation, and improve gel stability [15,16]. In addition, KGM effectively decreases the size of gelatinized starch granules and low field nuclear magnetic resonance experiments have confirmed that KGM can limit swelling and gelatinization of native starch and compete with corn starch for water [4,15]. Furthermore, KGM can increase the homogeneity and network density of pea and potato starch mixtures as determined by SEM (scanning electron microscopy), FT-IR (Fourier transform infrared) and differential scanning calorimetry (DSC). Many investigations have revealed the effect of KGM in maintaining texture and extending shelf life of starch-based food products [16,17]. However, the effect of KGM on pasting, paste, and gel properties of CS remains unknown.

The process of incorporating hydrocolloids into starch is known, but CS as a starch ingredient has yet to be fully investigated. In this study, we explored the properties of CS/KGM mixtures and their interactions on gelatinization, rheological, texture, microstructure, and thermal properties of blends with varied KGM concentrations. The findings from this study expand the applications of CS/KGM mixtures in food industries and provide guidance for development of CS-based food products.

## 2. Materials and Methods

### 2.1. Materials and Sample Preparation

CS (with an amylose concentration of approximately 22.5%) was supplied by Guangxi Tianlin Rongan Agricultural Development Co., Ltd. (Guangxi, China). KGMwas supplied by Seven Fairy Konjac Industrial Area Development Co., Ltd. (Hubei, China) The CS/KGM mixtures were prepared by dispersing CS and KGM (0, 0.3, 0.6, 0.9, and 1.2% dry basis) into distilled water with the total content of solids of 6%, *w*/*w*. First, CS was dispersed in distilled water under magnetic stirring, and then KGM was slowly added to CS solutions. The CS/KGM mixtures were gelatinized at 100 °C under magnetic stirring for 30 min. Rheological, texture, microstructure, particle size, and thermodynamic properties were all determined by this method.

### 2.2. Pasting Properties

The CS/KGM mixtures were prepared via dispersing weighed amounts of CS and KGM in deionized water (total solid content 6%, *w*/*w*, dry basis). Then, the mixtures were poured into aluminum canisters before insertion into a Rapid Visco Analyzer (RVA) (TecMaster, Perten Instruments, New South Wales, Australia). The heating and cooling cycles were programmed as follows: the mixtures were held at 50 °C for 1 min, heated to 95 °C at a constant rate of 6 °C/min, and held at 95 °C for 5 min, and subsequently cooled to 50 °C at a constant rate of 6 °C/min [18]. The stirring rate in the first 10 s was 960 rpm. Pasting properties were analyzed while maintaining a rotation speed of 160 rpm. The pasting curve was drawn by the instrument automatically.

### 2.3. Rheological Properties

Flow behavior and viscoelasticity of the freshly gelatinized CS/KGM dispersions obtained from the RVA were determined by using a rheometer (DHR-1, TA Instruments, New Castle, DE, USA) with a cone and plate geometry sensor (1 degree cone, 40 mm diameter, and 1 mm gap).

#### 2.3.1. Flow Behavior

The gelatinized CS/KGM dispersions were sheared stepwise from 0 to 300 s^−1^ followed by a decrease in shear rate from 300 to 0 s^−1^, for 630 s [16]. The data were fitted to the power law model described in Equation (1) as:

τ = K γ ^n−1^ (1)

where τ represents shear stress (Pa), K represents consistency index (Pa·s^n^), γ represents shear rate (s^−1^), and n represents flow behavior index.

#### 2.3.2. Frequency Sweep

For the dynamic viscoelasticity test, the linear viscoelastic region was measured at 25 °C using a strain sweep tests. The storage modulus (G′), loss modulus (G″), and tan δ were determined over the range of 0.1~10 Hz at 1% strain (determined in the linear region) [14]. 

#### 2.3.3. Oscillation Time Sweep

G′ and tan δ value of gelatinized CS and CS/KGM were monitored as a function of time with 1% strain amplitude at 0.5 Hz at 4 °C, for 1 h [19].

### 2.4. Texture Analysis

The CS/KGM mixtures, as described in Section 2.1, were cooled to room temperature (25 ± 1 °C) for 2 h, and then stored at 4 °C for 24 h in a refrigerator. The texture profile analysis (TPA) was conducted using a texture analyzer (CT3, Brookfield, Middleboro, MA, USA). Texture analysis of the mixtures was performed with a P/0.5 probe, at 1.0 mm/s pretest rate, 1.0 mm/s test rate, 1.0 mm/s return rate, 50% compression (approximately 5 mm test distance), and 5 g trigger force. The measurements were carried out in parallel six times [12].

### 2.5. Polarized Light Microscopy

Native CS and CS/KGM mixtures (1%, *w*/*v*) were measured at different temperatures (60, 70, and 95 °C) over 30 min period. One aliquot (about 20 µL) was placed directly on a clean microscope slide with a cover slip [20]. The starch granules were observed by a polarized light microscopy (BX43, Olympus, Tokyo, Japan) and pictures were captured at a magnification of 200×.

### 2.6. Scanning Electron Microscopy

The CS/KGM mixtures were uniformly coated in the culture dish, prefrozen at −18 °C for 24 h in the deep freezer (DW-40L278, Shandong., China), and then dried by vacuum freeze dryer (SCIENTZ-10ND, Ningbo, China) for 36 h, under 0.255 mbar vacuum, and panel temperature of −15 °C. The samples were deposited on copper stubs using double-sided adhesive tape and spraying ion sputtering metal. Then, the gel microstructure was observed by SEM (JSM-6510LV, JEOL, Tokyo, Japan) at an accelerating voltage of 15 kV under 200× magnification [21].

### 2.7. Differential Scanning Calorimetry (DSC)

The thermal properties of samples were carried out using DSC (DSC-Q2000, TA Instruments, Norwalk, CT, USA). Three milligrams of CS/KGM mixtures were loaded onto aluminum pans. Pans, after sealing hermetically, were allowed to equilibrate at 4 °C (24 h) to obtain a stable baseline, and then thermal analysis was performed in the temperature range from 30 to 90 °C with a heating rate of 10 °C/min under nitrogen atmosphere (flow rate of 20 mL/min) [14]. The onset temperature (To), peak temperature (Tp), end temperature (Tc), and endothermic enthalpy change (ΔH) were calculated. An aluminum pan without sample was used as the control, and each sample was repeated 3 times.

### 2.8. Statistical Analysis

All experiments were conducted in triplicate and data were reported as mean ± standard deviation. The analysis of data was performed using one-way analysis of variance (ANOVA) in SPSS 17.0 software (SPSS Korea, Data Solution, Seoul, Korea). Duncan’s new multiple-range test was used to determine the difference of means, and *p* < 0.05 was statistically significant.

## 3. Results and Discussion

### 3.1. Pasting Properties

The pasting characteristics and curves of the CS/KGM mixtures with various KGM concentrations are shown in Table 1 and Supplementary Figure S1. As the temperature increases from 50 to 95 °C, the pasting curves of CS in the presence and absence of KGM show significant variations (Supplementary Figure S1). The earlier slow increase in the viscosity of the CS/KGM mixture containing 1.2% KGM is caused by the dissolving of KGM (that is not rapidly soluble in cold water). KGM causes an earlier peak time (time to achieve the peak viscosity) as compared with the original CS. The presence of KGM increases the viscosity of the continuous phase, which is consistent with interactions between high amylose corn starch and hydrocolloids, due to the early increase in viscosity during the pasting process (lower pasting temperatures) [22]. However, no changes in peak time were observed with different KGM concentrations, which may be related to the molecular characteristic of KGM. It has been reported that different peak times were obtained among KGM, chitosan, and xanthan gum in corn resistant starch [22]. 

KGM added to CS resulted in increases in peak, breakdown, final, and setback viscosities. The mixtures show increased peak viscosities with an increasing percentage of KGM. At 1.2% KGM concentration, the peak viscosity (PV) of mixtures increases by 151.5% as compared with native CS. The high viscosities of the mixtures may be attributed to KGM’s strong ability for binding and absorbing water, which thereby causes less available water for starch hydration, increasing its local concentration and resulting peak viscosity [23,24,25]. This is consistent with the results reported by Liu et al. [24], in which the addition of edible gums (flaxseed gum and tamarind seed gum) positively affected PV. Furthermore, KGM addition exhibited large variations in the final viscosity (FV), from 1824 to 2598 mPa·s, which indicated that CS improved by KGM could be used as a thickener in foods. 

The breakdown viscosity (BV) reflects shear stability and resistance [26]. As shown in Table 1, CS has the lowest BV value of 1097 mPa·s, which increases by 123.2~267.4% for CS/KGM mixtures. It can be hypothesized from this result that the starch granules should become less resistant to mechanical shearing and easily rupture. This suggests that dissociations between starch and polysaccharide through the structural shrinkage of KGM due to a decrease in temperature could be responsible for the increase in the breakdown [27]. 

Pasting of CS begins at 68 °C, which is in accordance with the literature [5]. It is clearly seen from Supplementary Figure S1 that a rapid increase in viscosity (corresponding to pasting of starch component) takes place at exactly the same temperature for all samples. Decreases in the PT were observed with KGM concentrations from 0.9 to 1.2%, due to the rapid increase in viscosity of the CS/KGM system, which was detected by the RVA viscograph software. Additionally, the same decreasing trends have occurred in wheat starch with guar gum, tara gum, locust bean gum, and KGM [27]. 

### 3.2. Rheological Properties

#### 3.2.1. Flow Behaviors

**Figure 1:**
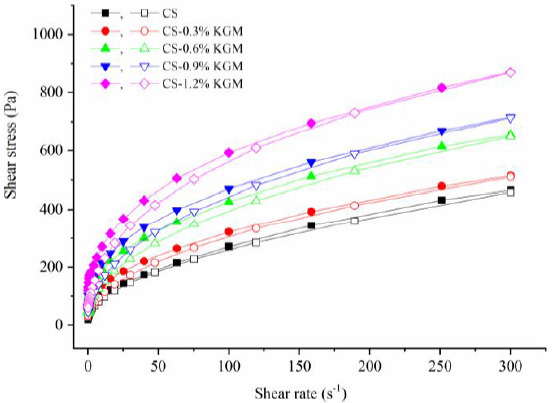
Flow curves of CS/KGM mixtures. Closed symbols denote the upward line and opened symbols denote the downward line.

Shear stress was plotted versus the shear rate for gelatinized CS with and without KGM at different concentrations (Figure 1). All samples had a pseudoplastic shear thinning behavior. The shear stress of the gelatinized CS/KGM dispersions increases with increasing KGM concentration from 0.3% to 1.2%, indicating that the adjunction of KGM can effectively influence flow behaviors. A previous report described a similar increase in shear stress of pearl millet with the addition of guar gum that was positively correlated with guar gum concentration [28].

The coefficient of determination (R^2^), flow behavior index (*n*), and consistency coefficient (K) are given in Table 2. All R^2^ are above 0.99, hence the model has high fitting precision. All mixtures showed pseudoplastic shear-thinning fluids, where n values were below 1. Meanwhile, the higher concentration of KGM (0.3~1.2%) exhibited with lower n values, was a decreasing trend in good agreement with those reported for corn starch/KGM [29], mung bean starch-xanthan/konjac gums [30], and rice starch/inulin mixtures [31]. We found that K values of mixtures were notably higher than the native CS and enhanced from 43.6 to 143.3 Pa·s^n^ with increasing KGM concentration (0.3~1.2%). This tendency is consistent with increasing pasting parameters in RVA measurements. Ma et al. presented a similar result that K values of corn starch/KGM mixtures were significantly enhanced from 3.15 to 28.78 Pa·s^n^ with increasing KGM concentration (0~0.3%) [14].

#### 3.2.2. Viscoelasticity

**Figure 2:**
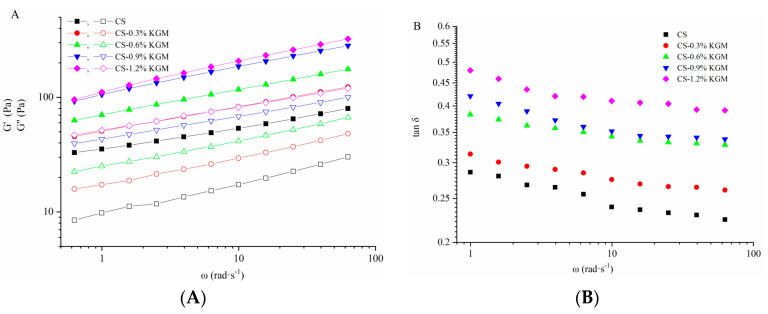
Curves of dynamic modylus (**A**) and tan δ (**B**) with angular frequency of CS/KGM mixtures. Closed symbols denote the storage modulus (G′) and opened symbols denote the loss modulus (G″).

Figure 2A shows G′ and G″ of gelatinized CS and CS/KGM mixtures at 25 °C in the frequency range of 0.1~10 Hz. All samples displayed typical biopolymer gel properties (G′ > G″), which indicated that elasticity played a dominant role [32]. G′ and G″ of CS/KGM mixtures were higher than CS and increased with KGM concentrations (0.3~1.2%). It is worth noting that KGM concentration reached 0.9% and 1.2%, respectively, and the increasing trend is more obvious. Simultaneously, a cross linkage network formatted via the presence of KGM further strengthens the elastic properties [25]. The increase in moduli (G′, G″) may attributed to promoted intermolecular associations of gelatinized starch in the presence of KGM [25,28]. Similar findings have been reported for wheat starch/KGM mixtures [27]. 

Tan δ value is commonly used to characterize the viscoelastic features of compound systems [33]. As shown in Figure 2B, KGM decreased the elasticity of CS (higher tan δ). The tan δ values of gelatinized CS/KGM dispersions increased with KGM concentration. These results reveal that the structure of CS/KGM dispersions become weaker and more liquid-like by adding KGM [33]. Similar results have been reported for non-ionic polysaccharides-wheat starch pastes which showed that KGM had a marked effect on increased tan δ value, and waxy rice starch-guar gum mixed pastes [27]. It was probably because of the interaction between KGM and amylose in the continuous phase, which retarded formation of intermolecular double helices of amylose and accordingly resulted in a less elastic structure. Further studies could focus on the interaction between KGM and amylose and its effect on rheological properties.

#### 3.2.3. Time-Dependent Rheological Properties

**Figure 3:**
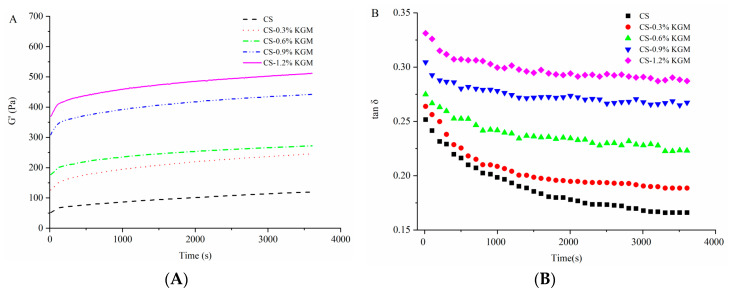
Curves of storage modulus (**A**) and tan δ (**B**) with time of CS/KGM mixtures.

Figure 3A shows the time dependence of G′ for CS pastes at different KGM concentrations. Thereafter, the G′ values increased slowly during storage for up to 1 h. The overall G′ values of CS/KGM dispersions were higher than the CS control and had a direct correlation with the KGM concentration. Similar research has been reported for maize starch pastes with or without the addition of guar gum [34]. As showed in Figure 3B, tan δ of gelatinized CS and CS/KGM dispersions decreased with time, suggesting the formation of a more elastic structure. Tan δ of gelatinized CS/KGM dispersions was higher than the CS control and had a positive correlation with KGM concentrations, which was in agreement with Figure 2B. KGM changed the gelatinized CS to a more viscous material, which is consistent with the pasting results in Section 3.1.

### 3.3. Textural Properties

The texture profile of starch gel is linked to its sensory properties [35]. The texture parameters, which include hardness, cohesiveness, chewiness, and elasticity of CS mixed with or without KGM were studied and the results are summarized in Table 3.

The hardness of gelatinized CS/KGM dispersions decrease markedly as compared with CS without KGM. The formation of amylose association contributes to the elastic gel network and promotes starch gel strength [36]. In the present study, a portion of CS was replaced by KGM to maintain the same total solid content, which accordingly reduced the concentration of amylose in the system. Thereby the hardness of CS/KGM gels decreased with the increased KGM concentration (decreased CS concentration). Similarly, Huang et al. reported that KGM did not cause an increase in the hardness of rice starch gel [37]. 

Table 3 shows that the cohesiveness, chewiness, and elasticity of starch gels increase significantly in the presence of KGM. Cohesiveness is a measure of gel setting and acceptability in starchy food. The CS gel exhibited lower cohesiveness than CS/KGM dispersions, which indicated severe texture damage. Incorporation of KGM to CS at a high level (1.2% *w*/*w*) increased the cohesiveness by 88.0%. Additionally, chewiness is a measure of the amount of energy required to masticate the starch-based gels. KGM (0.3~1.2%) increased chewiness values by 12.1~35.2% as compared with the CS control. The elasticity of CS/KGM gels were significantly higher than that of the CS gel and had a direct correlation with KGM concentration. The macromolecules interact with each other in CS/KGM systems which produces a matrix with enhanced three-dimensional network structure, with relatively high elasticity, cohesiveness, and chewiness. The obtained results show that the elasticities of samples significantly increase depending on the adjunction of KGM, especially at 0.9 and 1.2% KGM concentration, and the yield of elasticities increases by 39.1 and 53.3%, respectively, as compared with CS gel. Generally, the ability of starch gel to resist external damage is enhanced, which extends the application of CS, such as jelly-type deserts, noodles, and other starch-based products [38].

### 3.4. Morphological Structure of Starch Granules

**Figure 4:**
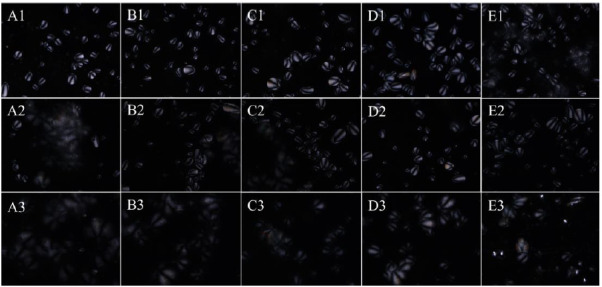
Polarized light microscopy of CS/KGM mixtures (200×). CS/KGM mixtures at different KGM concentrations (CS/0.3% KGM, 0.6% KGM, 0.9% KGM, and 1.2% KGM), were incubated at 60 °C (**A1**–**E1**), 70 °C (**A2**–**E2**), 95 °C (**A3**–**E3**).

Raw CS granules contain crystalline and show birefringence with the typical ”Maltese cross” under the polarization microscope. The loss of birefringence signal from the semi-crystalline areas occurs upon heating (from 50–95 °C) with the swell and destruction of starch granules. CS, a normal amylose-containing starch, is subjected to swelling in water at temperatures between 55 and 95 °C [39]. The effects of KGM on CS using polarized optical microscopy at 60 °C (limited swell of starch granules), 70 °C (close to the gelatinization temperature), and 95 °C (temperature starch granules swell extensively) [40], are showed in Figure 4. In addition, light microscopy experiment results are presented in Supplementary Figure S2, which show the gelatinization process of the CS and the CS/KGM dispersions [41].

CS granules have round or oval shapes and exhibit birefringence under polarized light at 60 °C. In addition, CS granules also have hilum, located at the edge of the round and oval starch granules. Differences in KGM concentration did not alter the granules morphology and Maltese crosses. Similar results have been previously reported [5].

At 70 °C, CS partially lost Maltese crosses and granular morphology, which illustrates destruction of the semi-crystalline structure in starch (Figure 4A2). The loss of Maltese crosses of CS at 70 °C was in agreement with gelatinization temperature of CS (68 °C, Section 3.1). At 95 °C, the CS was gelatinized (Section 3.1) accompanied by disruption of semi-crystalline structure and the absence of Maltese cross (Figure 4A3). The high temperature promoted transference of heat energy into the interior of starch granules, resulting in destruction of double helices structure [20]. KGM somewhat protected the inner semi-crystalline structure of CS from being destroyed by thermal treatment, and thereby partially kept the Maltese crosses at 70 °C (Figure 4B2–E2). At 95 °C, CS/KGM dispersions at a low KGM incorporation level (0.3% and 0.6%) lost the birefringence signal, but some starch granules showed Maltese cross in the dispersions at high KGM incorporation levels (0.9% and 1.2%). It is likely that KGM interacted with starch granules, which limited starch swelling and stabilized it from disruption at a high temperature [40,42]. KGM at a high level might limit the leak of amylose from swollen starch granules, and thereby result in the less elastic CS gels, as shown in Figure 2B. The relatively high G′ values of CS/KGM dispersions as compared with the CS control (Figure 2A) may be linked to the granular structure of some starch granules after heating, especially at high incorporation levels of KGM (Figure 4D3,E3). This may be useful for maintaining stability of starchy food during processing at a relatively high temperature.

### 3.5. Microstructure of Gelatinized Dispersions

**Figure 5:**
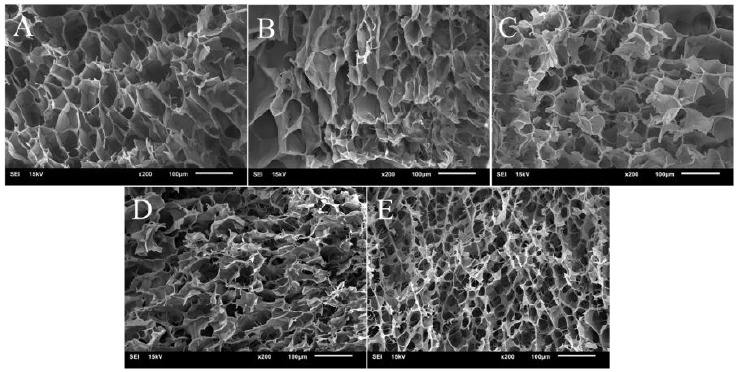
Microstructure of CS/KGM mixtures at different KGM concentrations. (**A**) CS; (**B**) 0.3% KGM; (**C**) 0.6% KGM, (**D**) 0.9% KGM; (**E**) 1.2% KGM.

The gelatinized CS and CS KGM dispersions displayed three-dimensional porous structures (Figure 5). The structure of gelatinized CS dispersion showed honeycomb-like structure with inhomogeneous sizes of holes and continuous lamellar connection between layers is observed. At a low KGM incorporation level (0.3 and 0.6%, Figure 5B,C), the morphology of the mixtures had no significant difference. This may be due to small amounts of KGM being present that did not induce a noticeable change in microstructure.

Upon further increase in KGM concentration (0.9% and 1.2%), the images show that the structure of CS/KGM mixtures display smaller homogeneous pore sizes and thicker gel skeleton structure. The thicker and denser gel skeleton structure may relate to the intermolecular interactions between KGM and CS, and formation of gels filling the matrix space between starch granule remnants [28]. With the addition of 1.2% KGM to CS, a filamentous network structure was observed (Figure 5E). A study has shown the appearance of similar filamentous network structure in high amylose corn starches with addition of guar gum and xanthan gum [22]. KGM may have a positive effect on promoting aggregation on amylose molecules in joining double-helical form [22].

### 3.6. Thermal Properties

Thermal properties of CS with the presence and absence of KGM were determined by DSC (Table 4). Gelatinization of CS shifted towards a higher temperature with the addition of KGM. The presence of KGM significantly increases Tp and Tc values of CS but decreases To and ΔH values of CS. KGM may influence CS gelatinization through competing water with starch, interacting with starch molecules, and limiting chain mobility of starch molecules [24,43]. The relatively high (Tc~To) values with the addition of KGM to CS may be attributed to the influence of KGM on molecular mobility in the system [43]. With the increases in KGM concentration (partial replacement of CS), ΔH values (calculated according to the total solid in the system) decreased, which was related to the decreased CS amount. In addition, the reduced ΔH with the presence of KGM may be related to the limited gelatinization of CS, as shown in Figure 5.

Thermal properties of CS with and without the presence of KGM agreed with the pasting properties (Table 1 and Supplementary Figure S1) and morphology of starch granules during heating (Figure 4). The early onset of viscosity with the addition of KGM to CS during pasting was linked to a decrease in To temperatures. The presence of KGM retarded starch gelatinization showing the remaining Maltese crosses at high temperatures (Figure 4) and increased gelatinization temperatures (Table 4).

## 4. Conclusions

The effect of KGM concentration on pasting, rheological, textural, thermal, and morphological properties of CS was assessed in this study. The addition of KGM to CS significantly increased pasting viscosities and G′ of gelatinized dispersions and as a result the material was more resistant to compression in the textural analysis. The presence of KGM protected the CS granules from thermal treatment and yielded the appearance of more Maltese crosses as compared with the CS control at 70 and 95 °C, indicating a decreased degree of gelatinization. The gelatinization temperatures of CS with the presence of different concentrations of KGM were significantly higher than the CS control. The addition of KGM to CS at a high concentration (1.2% *w*/*w*) resulted in the gel showing a denser structure with smaller cells and the formation of filamentous network structure. The findings of the present study advance the knowledge of interactions between KGM and CS and KGM’s influence on physicochemical and rheological properties. The CS/KGM mixture may find applications as a thickener and a stabilizer in many food products.

## Figures and Tables

**Table 1:** Pasting parameters of canna starch (CS)/konjac glucomannan (KGM) mixtures.

KGM Addition (%)	PV (mPa·s)	BV (mPa·s)	FV (mPa·s)	SV (mPa·s)	PT (°C)
0	2453 ± 128 ^e^	1097 ± 80 ^e^	1824 ± 272 ^c^	437 ± 17 ^b^	68 ± 1.5 ^a^
0.3	3935 ± 98 ^d^	2449 ± 176 ^d^	2070 ± 112 ^bc^	458 ± 24 ^b^	68 ± 1.5 ^a^
0.6	4580 ± 104 ^c^	2836 ± 225 ^c^	2181 ± 114 ^b^	468 ± 24 ^b^	67 ± 0.6 ^a^
0.9	5361 ± 336 ^b^	3522 ± 229 ^b^	2390 ± 196 ^ab^	551 ± 18 ^a^	63 ± 2.2 ^b^
1.2	6170 ± 181 ^a^	4030 ± 193 ^a^	2598 ± 179 ^a^	584 ± 7 ^a^	56 ± 1.6 ^c^

PV, peak viscosity; BV, breakdown viscosity; FV, final viscosity; SV, setback viscosity; PT, pasting temperature. Different letters in the same column indicate significant difference (*p* < 0.05).

**Table 2:** Flow properties for CS/KGM mixtures.

KGM Addition (%)	Up Curve			Down Curve		
	K (Pa·s^n^)	*n*	R^2^	K (Pa·s^n^)	*n*	R^2^
0	43.645 ± 2.162 ^e^	0.408 ± 0.013 ^a^	0.9964 ^b^	31.701 ± 2.232 ^e^	0.464 ± 0.020 ^a^	0.9947 ^e^
0.3	69.655 ± 3.314 ^d^	0.342 ± 0.004 ^b^	0.9938 ^e^	39.943 ± 3.043 ^d^	0.445 ± 0.004 ^b^	0.9956 ^d^
0.6	96.813 ± 5.021 ^c^	0.329 ± 0.001 ^c^	0.9949 ^d^	54.279 ± 4.821 ^c^	0.433 ± 0.003 ^c^	0.9967 ^c^
0.9	109.143 ± 7.364 ^b^	0.324 ± 0.001 ^d^	0.9956 ^c^	62.678 ± 5.326 ^b^	0.426 ± 0.001 ^d^	0.9969 ^b^
1.2	143.335 ± 9.634 ^a^	0.311 ± 0.001 ^e^	0.9997 ^a^	90.093 ± 5.867 ^a^	0.398 ± 0.001 ^e^	0.9973 ^a^

K, consistency coefficient; *n*, flow behavior index. Different letters in the same column indicate significant difference (*p* < 0.05) among various KGM concentrations.

**Table 3:** Parameters of texture profile of CS/KGM mixtures.

KGM Addition (%)	Hardness (N)	Cohesiveness	Chewiness (mJ)	Elasticity (mm)
0	3.86 ± 0.2 ^a^	0.25 ± 0.02 ^c^	9.1 ± 0.2 ^e^	9.2 ± 0.3 ^e^
0.3	3.23 ± 0.2 ^b^	0.39 ± 0.06 ^b^	10.2 ± 0.1 ^d^	10.1 ± 0.2 ^d^
0.6	2.71 ± 0.2 ^c^	0.40 ± 0.00 ^b^	10.8 ± 0.3 ^c^	11.2 ± 0.3 ^c^
0.9	2.33 ± 0.1 ^d^	0.46 ± 0.05 ^a^	11.6 ± 0.2 ^b^	12.8 ± 0.3 ^b^
1.2	1.99 ± 0.1 ^e^	0.47 ± 0.06 ^a^	12.3 ± 0.3 ^a^	14.1 ± 0.1 ^a^

Different letters in the same column indicate significant difference (*p* < 0.05) between various KGM concentrations.

**Table 4:** Thermal parameters of CS/KGM mixtures.

KGM Addition (%)	To (°C)	Tp (°C)	Tc (°C)	ΔH (J·g^−1^)	Tc-To (°C)
0	60.49 ± 0.21 ^a^	66.58 ± 0.01 ^c^	74.10 ± 0.01 ^e^	29.59 ± 0.01 ^a^	13.61 ± 1.05 ^e^
0.3	59.42 ± 0.15 ^a^	67.23 ± 0.02 ^b^	78.73 ± 0.01 ^c^	27.17 ± 0.01 ^b^	16.31 ± 1.02 ^d^
0.6	58.96 ± 0.92 ^a^	67.87 ± 0.01 ^b^	77.44 ± 0.00 ^d^	24.74 ± 0.02 ^c^	18.48 ± 0.87 ^c^
0.9	56.35 ± 0.82 ^b^	68.61 ± 0.02 ^a^	79.79 ± 0.01 ^b^	21.66 ± 0.07 ^d^	23.44 ± 0.76 ^b^
1.2	53.91 ± 0.62 ^c^	69.78 ± 0.01 ^a^	84.73 ± 0.01 ^a^	16.44 ± 0.08 ^e^	30.82 ± 0.57 ^a^

To, onset temperature; Tp, peak temperature; Te, end temperature; ΔH, endothermic enthalpy; Temperature range. Different letters in the same column indicate significant difference (*p* < 0.05) among various KGM concentrations.

## Supplementary Materials

The following are available online at https://www.mdpi.com/2304-8158/10/2/422/s1, Figure S1: Pasting curves of CS/KGM mixtures. Figure S2: Light microscopy micrographs (20×) of native CS and CS/KGM dispersions.
