# Supplementary material for: The Influence of Konjac Glucomannan on the Physicochemical and Rheological Properties and Microstructure of Canna Starch
Source: Foods. 2021 Feb 15;10(2):422. doi: 10.3390/foods10020422 (PMC7918958; doi:10.3390/foods10020422)
Supplement: Supplementary file 1 [file foods-10-00422-s001.pdf]

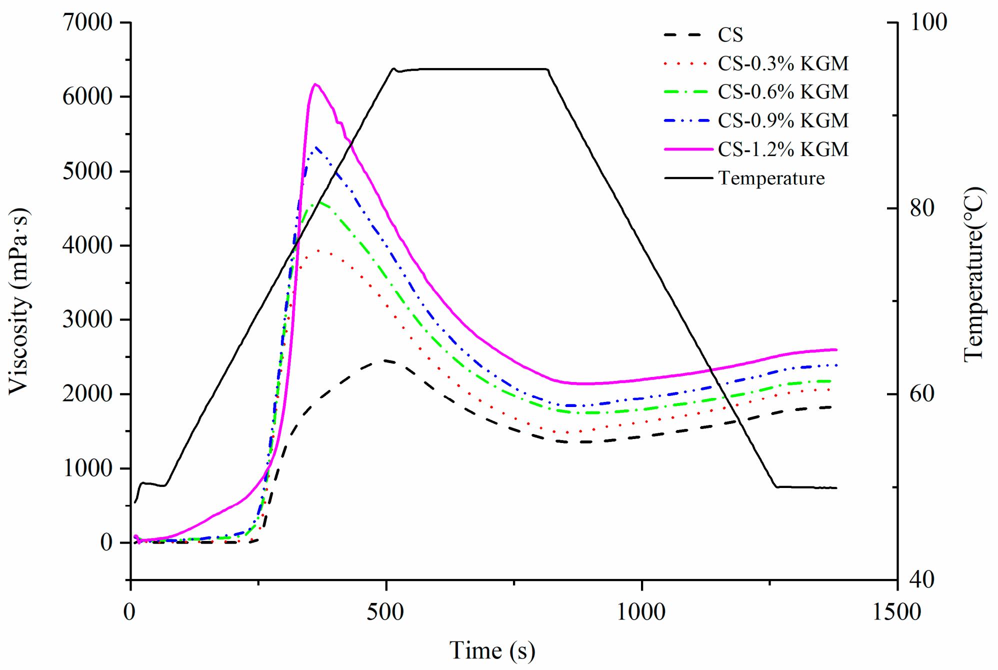

Figure S1: Pasting curves of CS/KGM mixtures.

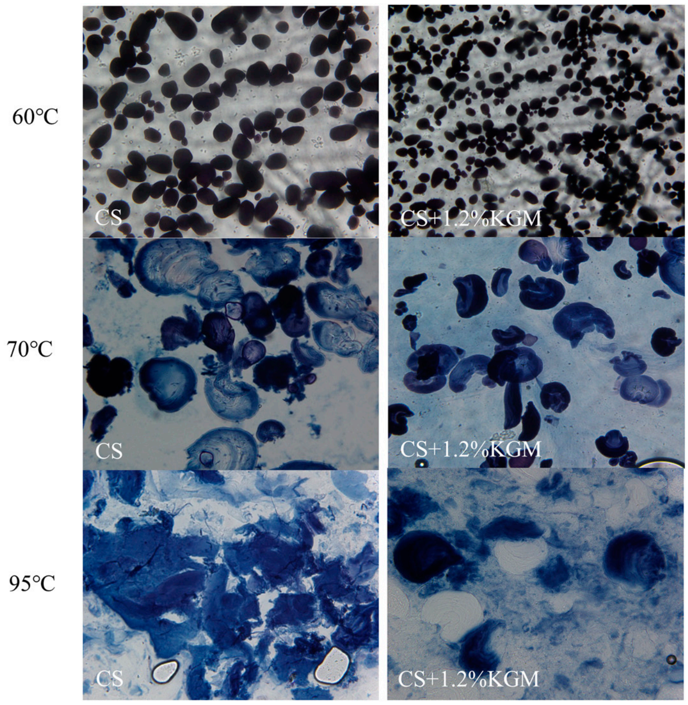

Figure S2: Light microscopy micrographs (20×) of native CS and CS/KGM dispersions.
